# Biomarker measurement in non-invasively sampled colorectal mucus as a novel approach to colorectal cancer detection: screening and triage implications

**DOI:** 10.1038/s41416-020-0893-8

**Published:** 2020-05-13

**Authors:** Alexandre Loktionov, Anet Soubieres, Tatiana Bandaletova, Nader Francis, Joanna Allison, Julian Sturt, Jai Mathur, Andrew Poullis

**Affiliations:** 1 DiagNodus Ltd, Babraham Research Campus, Cambridge, UK; 2 Department of Gastroenterology, St George’s Hospital, London, UK; 3 Department of Surgery, Yeovil District Hospital, Yeovil, UK; 4 Division of Surgery and Interventional Science, University College London, London, UK; 5 Department of Surgery, Southend University Hospital, Southend-on-Sea, UK; 6 Present Address: DiagNodus Ltd, St John’s Innovation Centre, Cowley Road, Cambridge, UK; 7 Present Address: Department of Gastroenterology, Charing Cross Hospital, London, UK

**Keywords:** Diagnostic markers, Colorectal cancer

## Abstract

**Background:**

Faecal tests are widely applied for colorectal cancer (CRC) screening and considered for triaging symptomatic patients with suspected CRC. However, faecal tests can be inconvenient, complex and expensive. Colorectal mucus (CM) sampled using our new patient-friendly non-invasive technique is rich in CRC biomarkers. This study aimed to evaluate diagnostic accuracy of CRC detection by measuring protein biomarkers in CM.

**Methods:**

Colorectal mucus samples were provided by 35 healthy controls, 62 CRC-free symptomatic patients and 40 CRC patients. Biomarkers were quantified by ELISA. Diagnostic performances of haemoglobin, C-reactive protein, tissue inhibitor of metalloproteinases-1, M2-pyruvate kinase, matrix metalloproteinase-9, peptidyl arginine deiminase-4, epidermal growth factor receptor, calprotectin and eosinophil-derived neurotoxin were assessed using receiver operating characteristic (ROC) curve analysis.

**Results:**

Colorectal mucus haemoglobin was superior compared to other biomarkers. For haemoglobin, the areas under the curve for discriminating between CRC and healthy groups (‘screening’) and between CRC and symptomatic patients (‘triage’) were 0.921 and 0.854 respectively. The sensitivity of 80.0% and specificities of 94.3% and 85.5% for the two settings respectively were obtained.

**Conclusions:**

Haemoglobin quantification in CM reliably detects CRC. This patient-friendly approach presents an attractive alternative to faecal immunochemical test; however, the two methods need to be directly compared in larger studies.

## Background

Colorectal cancer (CRC) is a global health problem, being the third most common cancer with 1,801,000 new CRC cases and 861,700 deaths worldwide in 2018.^1^ Importantly, it is predicted that CRC incidence worldwide will exceed 3 million by 2040.^2^ Slow tumour progression is a characteristic feature of sporadic CRC, which leaves ample time for its early diagnosis and curative treatment.^3^ However, colorectal tumours often do not cause any symptoms until advanced stages. In these circumstances, only effective CRC screening can substantially reduce mortality from CRC.^2,4,5^

Although full colonoscopy is widely regarded as the reference standard for CRC detection and is often employed for primary CRC screening,^5^ this diagnostic technique is not perfect, being invasive, expensive and sometimes causing complications. For this reason, two-step screening employing primary non-invasive testing followed by secondary colonoscopies only in positive cases remains the most popular CRC screening strategy all over the world.^2,4^

The traditional guaiac faecal occult blood test (gFOBT) had been used for non-invasive CRC detection for decades, but it has a very low sensitivity,^6^ and is currently being replaced with a more sensitive faecal immunochemical test (FIT), diagnostic sensitivity and specificity of which for CRC reach 74% and 95% respectively.^7^ In the USA, a recently introduced multitarget molecular stool test (‘Cologuard’)^8^ demonstrates an even higher sensitivity of 92.3% (at 89.8% specificity). However, the ‘Cologuard’ test requires whole stool collection^9^ and a complex multistep analytical procedure.^10^ At an extremely high cost of over $600 per assay, this test cannot be seriously considered for CRC screening. It appears that the combination of the highly cost-effective^11^ non-invasive FIT and confirmatory colonoscopies following positive FIT results can be regarded as the current strategy of choice for CRC screening.^12^

Population screening is a very important way of both detecting asymptomatic CRC cases and also preventing this cancer by pre-malignant polyp removal; however, it detects less than 20% of CRC cases diagnosed annually in the UK.^13^ The number of CRC cases diagnosed in England through the fast-track (2-week) referral pathway is considerably higher,^13,14^ but most colonoscopies performed in these symptomatic patients find neither CRC nor other serious colorectal conditions.^13^ The ‘lack of effective triage systems for invasive investigations’ is now highlighted as a critical research gap in the area of CRC.^15^ The introduction of the FIT for CRC triage in symptomatic patients is currently considered as a possible solution for this problem.^13,16^

Although faecal occult blood testing was successfully applied for several decades, the necessity of repeatedly collecting faeces for gFOBT^17^ often made it inconvenient and unpopular among screening participants,^18,19^ thus negatively affecting CRC screening uptake.^20–22^ The presently introduced FIT requires only a single faecal sample and was shown to improve screening uptake to 66.4%.^17^ However, this figure might be increased further if the necessity of collecting faeces could be eliminated.

It is now convincingly proven that all host cells and biomolecules that can be detected in human faeces are initially released from the normal or neoplastic mucosa and incorporated into the well-structured layer of colorectal mucus (CM)^23,24^ overlaying the mucosal surface.^25^ CM therefore presents the main repository of diagnostically informative biomarkers released from the colonic mucosa.^24^ Although its fragments are usually excreted with faeces, they are not uniformly distributed throughout the faecal matter; hence, diagnostic test performance may be compromised, especially when samples for the FIT are prepared by patients. Moreover, the bulk of the mucosa-associated CM permanently moves distally without being incorporated in the faeces and remaining on the mucosal surface.^24–26^ We have recently developed and clinically evaluated a new simple and patient-friendly technique for non-invasive CM collection^27^ suitable for cytological examination^28^ and diagnostic biomarker detection.^27,29^ This new approach was successfully used for inflammatory bowel disease (IBD) detection and monitoring.^29^ Recently it has also been tried as a CRC detection method in a small pilot project^30^ that allowed us to test 24 potential protein biomarkers of CRC and then to select nine best performers for further evaluation. The present study aimed to comparatively evaluate CRC detection efficiency by quantifying the selected protein biomarkers in CM for discriminating CRC cases from either healthy controls (‘screening’ setting) or cancer-free patients with abdominal symptoms (‘triage’ setting).

## Methods

### Study design and participants

The clinical part of this study included symptomatic patients with suspected CRC referred to have diagnostic colonoscopies and healthy volunteers, who were recruited at three clinical centres participating in the study: Department of Gastroenterology of St George’s Hospital (London, UK), Department of Surgery of Yeovil District Hospital (Yeovil, UK) and Department of Surgery of Southend University Hospital (Southend, UK). The protocol of the study was approved by London-South East Research Ethics Committee (16/LO/2273) in accordance with the Declaration of Helsinki. All recruited patients and healthy volunteers provided written informed consent. The project was supported by the NIHR Clinical Research Network and included in its Central Portfolio Management System (CPMS) under CPMS ID 33369. It was also registered as ISRCTN16782445.

**Fig. 1:**
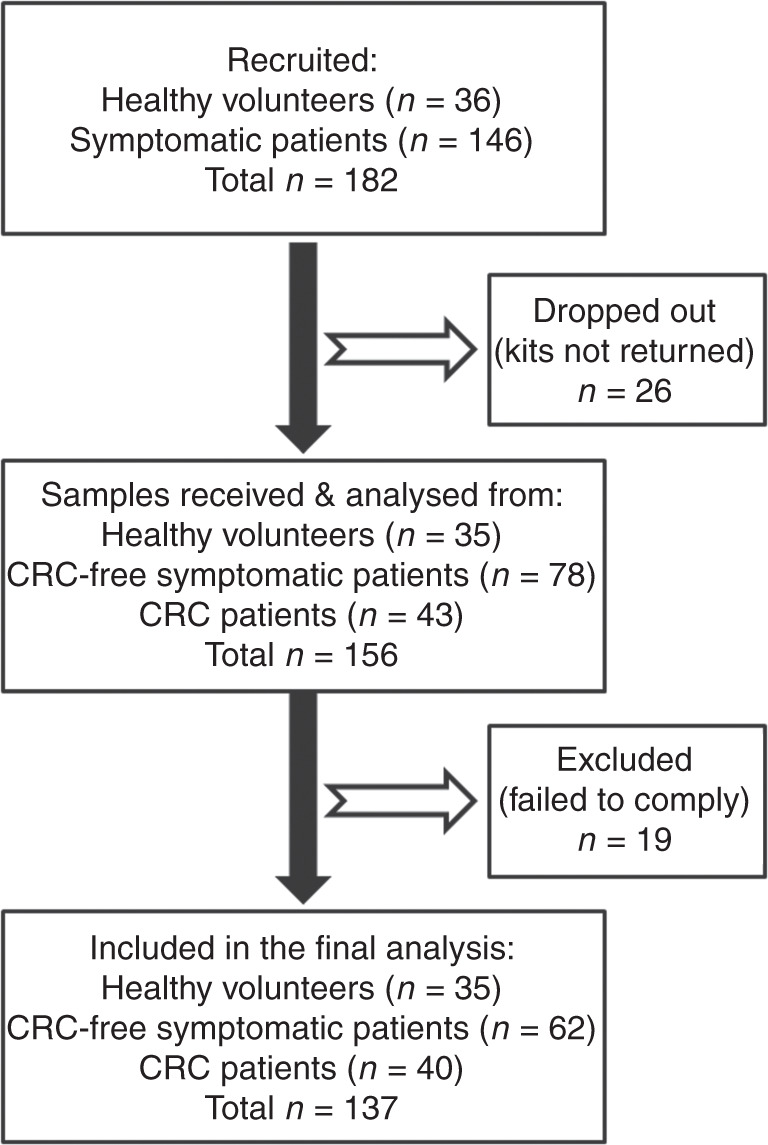
Study design and flowchart.

In this retrospective study, conducted between January 2017 and June 2018, clinically healthy volunteers and consecutive symptomatic patients were recruited. Symptomatic patients were enrolled following endoscopic investigation (often on the day of colonoscopy), and patients with diagnosed CRC provided samples before surgical intervention. Colonoscopy outcome was accepted as the diagnostic reference standard. Patients with concomitant inflammatory conditions (IBD, diverticulitis) or major colorectal surgery in the past were not considered for recruitment. Likewise, patients with colorectal polyps detected and removed during colonoscopy were not recruited. The required study size was estimated as at least 35 subjects per group required for detecting AUC difference of 0.150 at higher AUC = 0.850 with 95% confidence level and 80% power.^31^ Clinical study flowchart is presented in Fig. 1.

### CM sample collection

An original kit for non-invasive CM sampling (self-sampling) designed by DiagNodus Ltd and described in our previous publications^27–30^ was employed in this study. Briefly, samples were taken by swabbing the external anal area immediately following bowel opening using a swab coated with flocked nylon. The swabs were manufactured by Copan Flock Technologies (Brescia, Italy) according to the specifications defined by DiagNodus Ltd.

Each sampling kit comprised two swabs for sample collection, two polypropylene laboratory tubes containing 3 ml of (a) cell-preserving buffer^27^ and (b) cell lysis buffer,^27^ two microscope slides enveloped in a ‘slide card’, a small spray bottle containing cytology fixative, a set of instructions with a simple questionnaire and a pre-paid mailing envelope. All kit components were labelled using number codes. All samples were collected by study participants at home.

Two consecutive CM samples were obtained following one defaecation and prepared as follows: (1) the samples taken for biomarker analysis were immediately placed to the cell-preserving buffer; (2) two smears were prepared for cytological examination, and the residual material on the swabs was placed in the lysis buffer as described in our previous papers.^27–30^ Each study participant was requested to complete a simple brief questionnaire evaluating sampling procedure acceptability according to a 5-grade scale with answer options varying from 1—unacceptable to 5—convenient and comfortable.^30^ The collected samples and completed questionnaires were enclosed in the provided envelopes mailed directly to the laboratory of DiagNodus Ltd.

### Sample preparation and analysis

Before the analysis, the samples initially placed in the cell-preserving buffer were prepared, aliquoted and kept at −80 °C until use as previously described.^27,29,30^ Sample anonymity for blinded analysis was assured by the coded labelling. The samples initially placed in the cell lysis buffer were kept at 4 °C without any further preparation.

The microscope slides with smears were removed from the ‘slide cards’, and one slide from each pair was subjected to the conventional staining with haematoxylin and eosin. The remaining fixed smears were stored for possible further investigation. Stained smears were assessed microscopically by a highly experienced cytopathologist (T.B.) blinded to the clinical details. Descriptions of the stained smears reflecting the presence of different types of cells were prepared for all study participants.

### Biomarker quantification by enzyme-linked immunosorbent assays (ELISA)

Nine protein biomarkers selected in our preliminary study^30^ and comprising haemoglobin, C-reactive protein (CRP), tissue inhibitor of metalloproteinases-1 (TIMP1), M2-pyruvate kinase (M2-PK), matrix metalloproteinase-9 (MMP9), peptidyl arginine deiminase-4 (PADI4), epidermal growth factor receptor (EGFR), calprotectin and eosinophil-derived neurotoxin (EDN) were quantified in the collected CM samples using commercially available ELISA kits. ELISA kits for haemoglobin, CRP, TIMP1, MMP9 and EGFR were provided by Abcam (Cambridge, UK). M2-PK and PADI4 were detected using kits from MyBioSource (San Diego, CA, USA). Calprotectin was measured by kits from Calpro AS (Lysaker, Norway). Kits manufactured by MBL (Nagoya, Japan) were applied for the quantification of EDN. All ELISA assays were carried out according to the protocols provided by kit manufacturers, and calibration standards as well as quality controls supplied with the kits were used for assay calibration and examination quality testing. All standards and samples were analysed in duplicates. Sample dilutions used for obtaining optimal measurement results were as follows: M2-PK and PADI4—no dilution; TIMP1—1/4; EDN—1/5; haemoglobin and EGFR—1/10; calprotectin—1/50; MMP9—1/100; CRP—1/250. These optimal dilutions were determined in our previous study.^30^ Assay results were quantitatively measured using Multiscan FC plate reader (Thermo Fisher Scientific Oy, Vantaa, Finland), at absorbance wavelengths recommended by the ELISA kit manufacturers.

### Data analysis

Diagnostic information for all patients was collected from their clinical records. For CRC cases tumours were defined as proximal or distal according to their localisation proximally or distally from the splenic flexure, and tumour staging according to the TNM classification was recorded for each case.

ELISA result evaluation included the generation of calibration curves and absorbance measurement transformations into biomarker concentrations, which were then re-calculated for undiluted samples.

Statistical analyses were focused on assessing result distributions; however, descriptive statistics (means, standard deviations, standard errors, 95% confidence intervals, medians and ranges) were also calculated. Two-tailed Mann−Whitney test was applied to determine biomarker concentration differences between CRC patients, CRC-free symptomatic patients and healthy controls. Receiver operating characteristic (ROC) curve analyses were applied to evaluate biomarker performance for discriminating CRC cases from CRC-free symptomatic patients (‘triage’ setting) and CRC patients from healthy controls (‘screening’ setting). Areas under the curve (AUCs) were determined, and a recently proposed AUC-based method of optimal cut-off point determination^32^ was employed for determining biomarker-specific cut-off points (values obtained for the ‘triage’ setting were used). Sensitivity and specificity values for CRC detection were then determined. Quantitative data handling and statistical analyses were performed using IBM SPSS 19.0 statistical package (IBM Inc., Armonk, NY, USA). Diagnostic Accuracy Studies (STARD 2015) guidelines^33,34^ were followed for manuscript preparation.

## Results

### General characteristics of study participants

Figure 1 shows that sampling kits were given to 36 healthy volunteers and 146 symptomatic patients. CM samples were successfully collected and sent to the laboratory by 35 healthy volunteers (97.2% of the recruited volunteers) and 121 symptomatic patients (82.9% of the recruited patients). As most of the symptomatic patients were recruited on the day of endoscopy, they were instructed not to collect samples within at least 48 h following colonoscopy, but 11 patients disregarded this recommendation. Five patients had active inflammation (diverticulitis) at the time of sampling and three patients had major colorectal surgery in the past (not disclosed during recruitment). All these 19 patients were excluded from the analysis (see Fig. 1). The final number of eligible study participants was 137, including 35 control healthy volunteers (19 males and 16 females, age range: 17−56 years, median age: 38 years), 62 CRC-free symptomatic patients (33 males and 29 females, age range: 21−84 years, median age: 65.5 years) and 40 CRC patients (27 males and 13 females, age range: 23−93 years, median age: 68 years). In the symptomatic CRC-free group, there were 16 cases of diverticulosis and 13 cases of haemorrhoids. Fourteen patients of this group had small polyps (not removed during endoscopy) and 12 had irritable bowel syndrome (IBS) diagnosis. In the CRC group, there were 17 proximal and 23 distal tumours. Among them, 36 tumours were histopathologically diagnosed as adenocarcinomas and four as mucinous carcinomas (see Table 1). The following distribution of cases according to the TNM classification was observed: Stage I—6; Stage IIA—13; Stage IIB—2; Stage IIIA—1; Stage IIIB—8; Stage IIIC—9; Stage IVA—1 (see details in Table 1).

**Table 1:** Individual results of measuring protein biomarkers in colorectal mucus samples non-invasively collected from CRC patients.

#	Age (years)	Sex	CRC site	CRC histology	TNM stage	Haemoglobin (ng/ml)	CRP (ng/ml)	TIMP1 (ng/ml)	M2-PK (U/ml)	MMP9 (ng/ml)	PADI4 (ng/ml)	EGFR (pg/ml)	Calpro (μg/ml)	EDN (ng/ml)
Proximal tumours														
1	59	F	Asc	AdCa	SI(T1N0M0)	0.0	0.0	1.7	2.2	**18.9**	**1.2**	**511.4**	1.0	3.8
2	74	M	Asc	AdCa	SI(T2N0M0)	69.7	**11.1**	**5.9**	**22.3**	7.3	**2.5**	**375.7**	2.3	5.8
3	75	F	Trans	AdCa	SI(T2N0M0)	**182.6**	0.0	1.0	0.8	**19.8**	**1.3**	208.8	2.1	6.6
4	93	M	Hep fl	AdCa	SIIA(T3N0M0)	**490.3**	0.0	**3.6**	**28.0**	**14.8**	**12.3**	**341.7**	**5.1**	1.5
5	72	M	Trans	AdCa	SIIA(T3N0M0)	>**5000.0**	**535.8**	**12.3**	**19.0**	**54.5**	**1.3**	**583.0**	**9.8**	**19.9**
6^a^	23	F	Caecum	AdCa	SIIA(T3N0M0)	**4855.2**	**96.8**	**8.3**	**12.7**	**40.9**	**1.4**	**471.5**	**15.3**	**39.7**
7	71	M	Trans	AdCa	SIIA(T3N0M0)	**239.6**	**14.6**	**11.0**	4.9	**10.7**	0.5	**544.4**	**4.3**	4.9
8	73	M	Asc	AdCa	SIIIA(T2N1M0)	**991.4**	**17.7**	**7.4**	**22.7**	**44.0**	**2.9**	**547.4**	**14.0**	4.9
9	90	F	Trans	Muc Ca	SIIIB(T3N1M0)	0.0	2.5	**15.5**	**12.8**	5.2	**1.4**	291.2	0.4	4.6
10	64	F	Caecum	AdCa	SIIIB(T3N1M0)	**939.3**	2.7	0.8	3.0	0.4	0.2	75.8	0.6	**7.1**
11	60	M	Caecum	AdCa	SIIIB(T3N1M0)	12.2	1.1	1.0	0.5	2.7	0.0	178.4	1.7	1.3
12	76	M	Caecum	AdCa	SIIIB(T3N1M0)	**2883.6**	**39.8**	1.5	0.8	5.4	0.9	165.8	0.8	**20.3**
13	67	M	Trans	AdCa	SIIIB(T3N1M0)	**2879.2**	**24.1**	**16.6**	**15.7**	**26.4**	**2.0**	**329.5**	**4.7**	**33.7**
14	69	M	Trans	AdCa	SIIIC(T3N2M0)	**2646.3**	**26.1**	**10.1**	**58.3**	**50.2**	**6.0**	**1456.0**	**21.0**	**25.2**
15	52	M	Caecum	AdCa	SIIIC(T3N2M0)	0.0	**28.0**	0.3	3.6	3.5	0.3	41.8	0.2	1.3
16	60	M	Trans	AdCa	SIIIC(T3N2M0)	0.0	**17.2**	0.8	0.0	5.4	0.2	13.6	0.0	1.3
17	78	F	Caecum	AdCa	SIIIC(T3N2M0)	**1896.4**	**15.9**	**8.2**	**11.2**	**41.3**	**5.0**	103.1	**3.4**	9.9
DIstal tumours														
18	64	M	Rectum	AdCa	SI(T1N0M0)	>**5000.0**	**20.9**	1.5	4.4	1.9	0.3	158.8	0.6	1.9
19	60	M	Rectum	AdCa	SI(T2N0M0)	>**5000.0**	**352.3**	**12.5**	**11.2**	**23.2**	**1.6**	**1076.5**	**17.6**	**39.5**
20	64	M	Sigmoid	AdCa	SI(T2N0M0)	**745.5**	**18.9**	**4.8**	1.3	**22.1**	0.9	**328.9**	2.5	8.1
21	81	F	Rectum	AdCa	SIIA(T3N0M0)	>**5000.0**	**66.3**	**16.6**	**95.8**	**262.3**	**23.2**	**954.9**	**12.4**	**105.6**
22	63	M	Sigmoid	AdCa	SIIA(T3N0M0)	>**5000.0**	**59.0**	**>120.0**	**46.0**	**97.3**	**7.4**	**1164.0**	**26.4**	**13.5**
23	65	F	Rectum	AdCa	SIIA(T3N0M0)	**215.5**	1.3	**4.7**	**9.9**	**17.3**	**1.3**	73.1	0.3	6.9
24	84	M	Rectum	AdCa	SIIA(T3N0M0)	>**5000.0**	**60.4**	**99.7**	**39.3**	**26.4**	**6.7**	**417.5**	**5.2**	7.8
25	78	M	Rectum	Muc Ca	SIIA(T3N0M0)	>**5000.0**	**40.5**	0.4	2.1	1.5	0.1	80.3	2.1	3.4
26	60	M	Sigmoid	AdCa	SIIA(T3N0M0)	>**5000.0**	**66.6**	**30.0**	**42.4**	**117.6**	**12.3**	193.5	**9.5**	**20.4**
27	66	M	Rectum	AdCa	SIIA(T3N0M0)	**4897.3**	**101.8**	**27.8**	**32.5**	**37.7**	**5.6**	101.8	**4.1**	6.0
28	59	M	Desc	AdCa	SIIA(T3N0M0)	**124.1**	**285.7**	**37.6**	**28.3**	**52.1**	**3.2**	285.7	**9.2**	**24.0**
29	70	M	Sigmoid	AdCa	SIIA(T3N0M0)	**1521.1**	**23.2**	**11.2**	2.8	**65.5**	**2.0**	**343.7**	**15.9**	**22.2**
30	72	F	Rectum	Muc Ca	SIIB(T4N0M0)	**295.91**	**11.6**	2.1	**9.1**	6.4	0.9	**1180.5**	**18.4**	**13.3**
31	81	M	Rectum	AdCa	SIIB(T4N0M0)	>**5000.0**	**85.7**	**14.7**	**67.5**	**64.8**	**11.1**	**310.5**	**8.4**	**53.5**
32	60	M	Rectum	AdCa	SIIIB(T3N1M0)	>**5000.0**	**160.3**	**>120.0**	**54.0**	**266.2**	**8.2**	**1069.9**	**22.8**	**32.3**
33	69	F	Rectum	AdCa	SIIIB(T3N1M0)	40.6	7.2	0.3	3.4	9.9	0.1	8.0	0.0	1.5
34	55	F	Sigmoid	AdCa	SIIIB(T3N1M0)	**152.8**	6.7	**3.3**	**25.1**	4.5	**3.5**	**4771.8**	**3.9**	**28.7**
35	71	F	Sigmoid	AdCa	SIIIC(T3N2M0)	4.0	**10.8**	0.9	1.2	1.0	0.0	88.5	0.0	8.0
36	69	M	Sigmoid	AdCa	SIIIC(T3N2M0)	>**5000.0**	**31.5**	**>120.0**	**54.8**	**325.3**	**8.7**	**1242.1**	**22.9**	**23.2**
37	60	M	Sigmoid	AdCa	SIIIC(T3N2M0)	>**5000.0**	**64.4**	**68.4**	**12.9**	**134.6**	**1.2**	**464.0**	**144.5**	**17.3**
38	36	F	Sigmoid	Muc Ca	SIIIC(T4N2M0)	**220.0**	5.8	0.6	0.0	1.8	0.2	44.5	0.0	4.4
39	47	M	Sigmoid	AdCa	SIIIC(T4N2M0)	>**5000.0**	**160.4**	**>120.0**	**56.5**	**356.9**	**14.3**	**2248.2**	**22.0**	**226.7**
40	69	M	Rectum	AdCa	SIVA(T3N1M1)	**4217.9**	**14.7**	**11.0**	**10.4**	**11.6**	**3.3**	270.3	1.3	5.2

Results are sorted according to case severity (TNM staging). Biomarker test results shown in bold are positive.

*AdCa* adenocarcinoma, *Asc* ascending colon, *Desc* descending colon, *F* female, *Hep Fl* hepatic flexure, *M* male, *Muc Ca* mucinous carcinoma, *Trans* transverse colon.

^a^Lynch syndrome suspected in this case.

### Sampling quality assessment by study participants

Completed questionnaires were returned by 31 healthy volunteers and 108 symptomatic patients (comprising 38 CRC cases). None of the study participants reported any problems related to sample collection. Comparison between study groups has not revealed significant differences in sampling quality assessment; therefore, total estimate for the 139 respondents was calculated. The resulting average grade of 4.45 (95% CI between 4.32 and 4.57) was between the predetermined grades 5 (convenient & comfortable) and 4 (acceptable—OK). The average time required for sampling procedure completion was 6.2 min (95% CI between 5.3 and 7.1 min).

### Cytological analysis of CM smears

All 35 CM smears from healthy volunteers could be analysed cytologically. These smears usually contained very few cells (exfoliated normal colonocytes and occasional neutrophils).

In the group of 62 symptomatic CRC-free patients, samples from 51 subjects were suitable for cytology (ten smears were poorly prepared; two smears were too heavily contaminated for cytological examination). A range of cytological manifestations was observed in this diverse group. Neutrophils were present in 11 cases, eosinophils in 6 cases, erythrocytes in 12 cases and apoptotic bodies in 6 cases. Haematoxylin-positive fibre-like structures interpreted as manifestations of extracellular DNA trap formation (ET-osis) were noted in 12 smears. Exfoliated normal colonocytes were present in 40 cases.

**Fig. 2:**
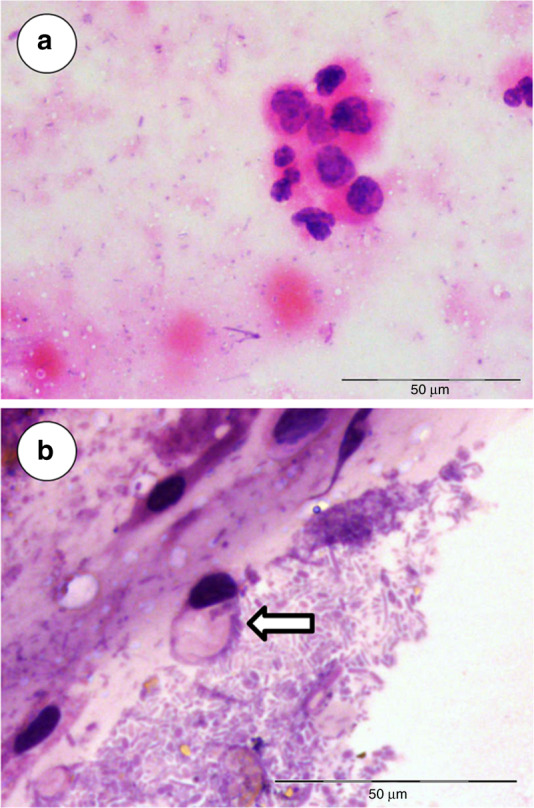
The presence of malignant cells in CM samples collected from CRC patients (haematoxylin & eosin stain). **a** A cluster of exfoliated malignant cells originating from a moderately differentiated rectal adenocarcinoma (see CRC case #21 in Table 1). **b** An exfoliated ‘signet ring’ cell (arrow) originating from transverse colon mucinous carcinoma (see CRC case #9 in Table 1).

Cytological analysis of 35 CM samples from 40 eligible CRC patients could be performed. In five cases smears were poorly prepared and unreadable. Distinctly identifiable tumour cells could be seen in smears from 11 CRC patients (see Fig. 2). Neutrophil presence was relatively common (19 cases). Erythrocytes were detected in 16 smears, and apoptotic bodies were observed in 15 cases. Signs of ET-osis were found in 12 cases, typically alongside neutrophils. The presence of exfoliated normal colonocytes was observed in CM samples from 28 CRC patients. It should be noted that only the presence of cancer cells could be regarded as diagnostically conclusive; thus, cytological CRC detection in 11 out of 35 analysed samples corresponds to the sensitivity of only 31.4%.

### Diagnostic performance of CRC biomarkers

**Fig. 3:**
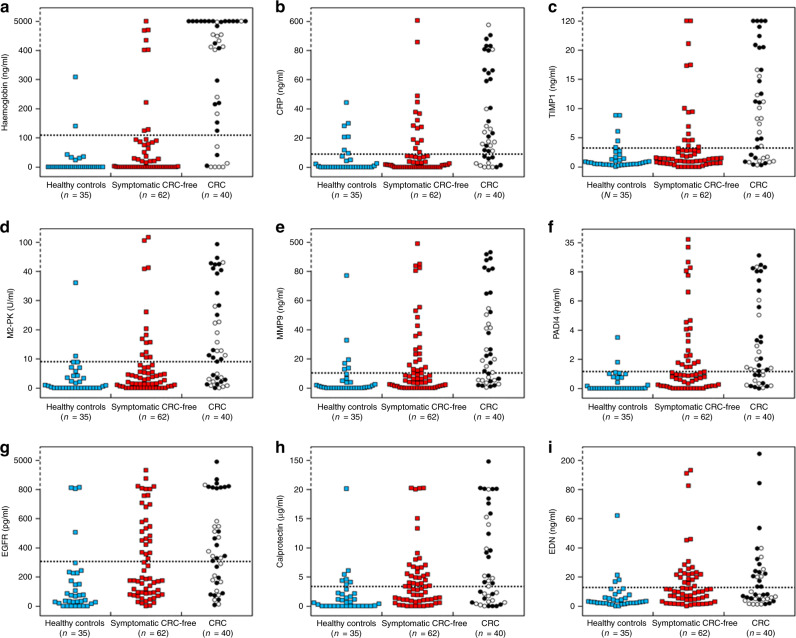
Distributions of individual results for haemoglobin (**a**), CRP (**b**), TIMP1 (**c**), M2-PK (**d**), MMP9 (**e**), PADI4 (**f**), EGFR (**g**), calprotectin (**h**) and EDN (**i**) in healthy controls (blue squares), cancer-free symptomatic patients (red squares) and CRC patients (circles). In the CRC group: light symbols—proximal tumours; dark symbols—distal tumours.

**Fig. 4:**
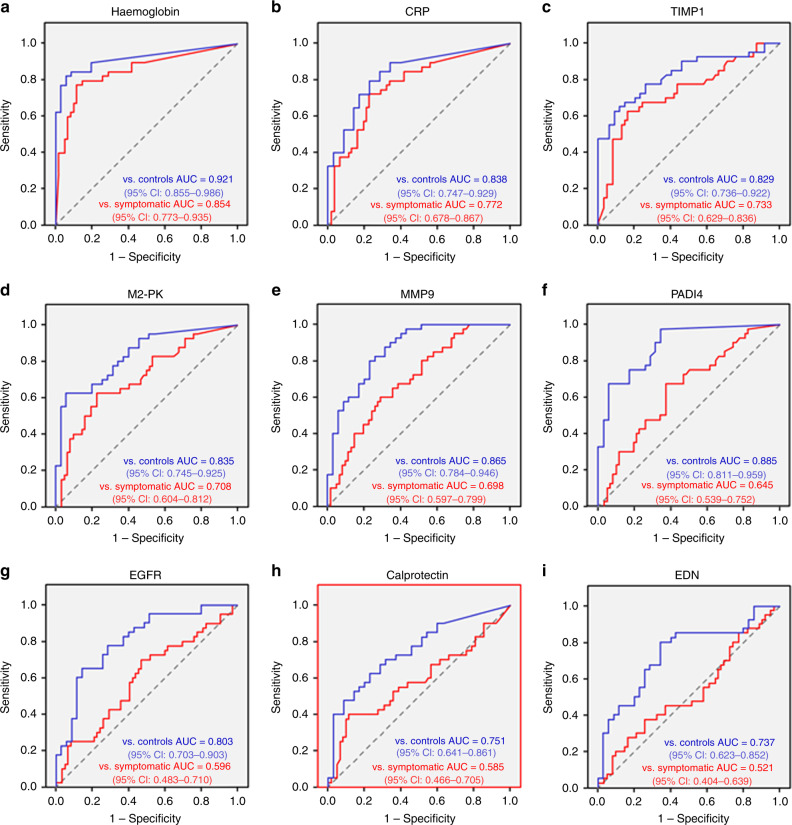
ROC curves for ‘screening’ (blue) and ‘triage’ (red) settings for haemoglobin (**a**), CRP (**b**), TIMP1 (**c**), M2-PK (**d**), MMP9 (**e**), PADI4 (**f**), EGFR (**g**), calprotectin (**h**) and EDN (**i**).

Results of protein biomarker quantification in non-invasively collected CM samples are presented in Table 2 and Figs. 3 and 4. Separate ROC curves were generated for comparing CRC cases with either healthy controls (‘screening’ setting) or cancer-free symptomatic patients (‘triage’ setting). Haemoglobin was clearly the best performer in both the settings, producing AUCs of 0.921 (95% CI between 0.855 and 0.986) and 0.854 (95% CI between 0773 and 0.935) for the ‘screening’ and ‘triage’ scenarios respectively (Fig. 4a). It is also evident that in the ‘screening’ setting such markers as CRP, TIMP1, M2-PK, MMP9 and PADI4 demonstrated relatively high AUC values (between 0.829 and 0.885), whereas AUC values over 0.700 for CRC ‘triage’ were observed only for CRP, TIMP1 and M2-PK (Fig. 4). EGFR, calprotectin and EDN could discriminate between CRC patients and healthy controls (AUCs between 0.737 and 0.803), however, failed to perform reliably in the ‘triage’ setting (Fig. 4). When test sensitivity and specificity values were calculated for all biomarkers, the diagnostic sensitivity of haemoglobin reached 80.0% (95% CI between 63.4% and 90.4%) at the specificity of 94.3% (95% CI between 79.5% and 99.0%) for the ‘screening’ setting and 85.5% (95% CI between 73.7% and 92.7%) for the ‘triage’ setting (see Table 2). All other tested biomarkers clearly had lower sensitivity and specificity values, especially in the ‘triage’ setting (Table 2). It is also important to stress that CM sample testing for haemoglobin resulted in the clustering of perfectly negative (no marker detected) results for most samples taken from either healthy controls or cancer-free symptomatic patients. In contrast, strongly positive (over 500 ng/ml) results were observed in most CRC cases (see Fig. 3a and Table 1). Occasional false-negative results, however, occurred in the latter group. Given that in this study CM samples were collected only once, it is impossible to exclude that some patients could fail to collect sufficient amounts of CM. This problem seemed to provide a likely explanation for consistently low results for all biomarkers in patients #11 and #33 (see Table 1), but tumour location could be another factor since biomarker concentrations detected in patients with proximal CRC tended to be generally lower (see Fig. 3). Likewise, proximal CRC was certainly associated with higher numbers of false-negative results (Fig. 3).

**Table 2:** Comparison of tested colorectal mucus biomarker performance for CRC detection vs. groups of asymptomatic healthy volunteers (controls) and patients with abdominal symptoms (based upon ROC curve analysis).

Biomarker	Optimal cut-off level	Sensitivity (%) [95% CI]	AUC vs. control [95% CI]	Specificity vs. control (%) [95% CI]	AUC vs. Sympt. Pat-s [95% CI]	Specificity vs. Sympt. Pat-s (%) [95% CI]	Median biomarker conc. (Control)	Median biomarker conc. (Sympt. Pat-s)	Median biomarker level (CRC)
Haemoglobin	109.3 ng/ml	80.0 [63.4−90.4]	0.921 [0.855−0.986]	94.3 [79.5−99.0]	0.854 [0.773−0.935]	85.5 [73.7−92.7]	0.0 ng/ml^a, b^	0.0 ng/ml^a, c^	1708.7 ng/ml^b, c^
CRP	8.9 ng/ml	72.5 [55.9−84.9]	0.838 [0.747−0.929]	80.0 [62.5−90.9]	0.772 [0.678−0.867]	75.8 [63.0−85.4]	0.0 ng/ml^d^	1.4 ng/ml^e^	22.1 ng/ml^d, e^
TIMP1	3.2 ng/ml	67.5 [50.8−80.9]	0.829 [0.736−0.922]	85.7 [69.0−94.6]	0.733 [0.629−0.836]	75.8 [63.0−85.4]	0.7 ng/ml^f, g^	1.4 ng/ml^f,h^	8.3 ng/ml^g, h^
M2-PK	9.0 U/ml	62.5 [45.8−76.8]	0.835 [0.745−0.925]	94.3 [79.5−99.0]	0.708 [0.604−0.812]	77.4 [64.7−86.7]	0.6 U/ml^i, j^	3.0 U/ml^i,k^	12.0 U/ml^j, k^
MMP9	10.4 ng/ml	65.0 [48.3−78.9]	0.865 [0.784−0.946]	82.9 [65.7−92.8]	0.698 [0.597−0.799]	64.5 [51.3−76.0]	0.4 ng/ml^l, m^	6.4 ng/ml^l,n^	20.9 ng/ml^m, n^
PADI4	1.2 ng/ml	67.5 [50.8−80.9]	0.885 [0.811−0.959]	94.3 [79.5−99.0]	0.645 [0.539−0.752]	62.9 [49.7−74.6]	0.0 ng/ml^o, p^	0.9 ng/ml^o,q^	1.5 ng/ml^p, q^
EGFR	305.5 pg/ml	60.0 [43.4−74.7]	0.803 [0.703−0.903]	88.6 [72.3−96.3]	0.596 [0.483−0.710]	58.1 [44.9−70.3]	67.7 pg/ml^r, s^	187.0 pg/ml^r^	342.7 pg/ml^s^
Calprotectin	3.4 µg/ml	57.5 [41.0−72.6]	0.751 [0.641−0.861]	80.0 [62.5−90.9]	0.585 [0.466−0.705]	56.5 [43.3−68.8]	0.6 µg/ml^t, u^	2.9 µg/ml^t^	4.0 µg/ml^u^
EDN	12.8 ng/ml	45.0 [29.6−61.3]	0.737 [0.623−0.852]	88.6 [72.3−96.3]	0.521 [0.404−0.639]	62.9 [49.7−74.6]	2.8 ng/ml^v, w^	10.1 ng/ml^v^	8.0 ng/ml^w^

*P* values for CM biomarker level comparisons between study groups:

^a^*P* < 0.01878; ^b^*P* < 0.00001; ^c^*P* < 0.00001; ^d^*P* < 0.00001; ^e^*P* < 0.00001; ^f^*P* = 0.02640; ^g^*P* < 0.00001; ^h^*P* = 0.00008; ^i^*P* = 0.01352; ^j^*P* < 0.00001; ^k^*P* = 0.00040; ^l^*P* = 0.00288; ^m^*P* < 0.00001; ^n^*P* = 0.00078; ^o^*P* < 0.00001; ^p^*P* < 0.00001; ^q^*P* = 0.01428; ^r^*P* = 0.00020; ^s^*P* < 0.00001; ^t^*P* = 0.00042; ^u^*P* = 0.00020; ^v^*P* = 0.00064; ^w^*P* = 0.00044.

## Discussion

The introduction of non-invasive biomarker-based tests to triage patients with abdominal symptoms for selecting those requiring immediate endoscopic investigations is an unmet clinical need.^13,15^ Our recent work focused on CM has demonstrated that CM sample collection is a simple procedure very well accepted by patients.^27,30^ In our previous preliminary study we tested 24 proteins detectable in CM and regarded as potential CRC biomarkers and selected nine best performers for further evaluation.^30^ The outcome of the present study confirms our preliminary findings and clearly identifies haemoglobin as the most reliable CRC marker present in CM samples. The values of CM haemoglobin test sensitivity of 80.0% (95% CI between 63.4% and 90.4%) and specificity of 94.3% (95% CI between 79.5% and 99.0%) for the ‘screening’ setting obtained in this study were similar or slightly higher compared to those reported for CRC screening by FIT.^7^ It is also important to stress that in the ‘triage’ setting the 85.5% (95% CI between 73.7% and 92.7%) specificity of testing haemoglobin in CM was only slightly lower than for our ‘screening’ setting. This point is important since none of the other biomarkers evaluated in this study could reach 80% specificity in the ‘triage’ setting.

In addition to showing an impressive diagnostic performance of CM haemoglobin for CRC detection, the present study has reproduced our earlier findings, further confirming the non-invasive CM sampling procedure to be very patient-friendly, as feedback from study participants convincingly demonstrated. Even in its more complex version that comprised preparation of smears for cytology, which will not be needed for the clinical use of the test, the average duration of the sampling procedure was only 6.2 min. Moreover, several study participants apparently had experience of collecting material for stool testing in the past and typically commented that the new test was ‘much simpler and easier than the old one’. These observations indicate that non-invasive testing of CM samples for haemoglobin can potentially present an attractive alternative to the FIT. CM sampling kit is very simple, and immunochemical CM sample testing for haemoglobin differs very little from faecal sample testing. It is therefore obvious that the cost of the CM test will be similar to that of the FIT, which is currently regarded as the optimal method for non-invasive CRC screening in terms of cost-effectiveness.^11^

In the beginning of this project we did not expect to identify haemoglobin as the best CRC biomarker amongst proteins present in the CM, but the presented results clearly show that all other tested biomarkers were less efficient in detecting CRC. Surprisingly, CRP has emerged as the second-best performing biomarker in the ‘triage’ setting. Elevated serum CRP concentrations are known to be associated with active IBD^35^ and were reported to correlate with CRC-caused mortality,^36^ but the presence of this protein in the faeces of CRC patients has not been investigated so far. Our results on CRC-associated increase of this inflammation-related protein in the CM look intriguing, especially in view of poor diagnostic performance of other inflammation-related proteins (calprotectin and EDN). However, it should be noted that for reliable CRP quantification CM samples need to be diluted 1/250, which makes this assay less convenient practically. TIMP1 diagnostic performance appeared to be slightly inferior compared to that of CRP. This protein closely involved in extracellular matrix remodelling is known to be upregulated in colorectal tumours^37–39^ and was demonstrated to be involved in metastasis-associated angiogenesis.^38^ In addition, TIMP1 is regarded as a plasma or serum biomarker of CRC^40–42^ and was shown to be frequently present in stool samples from CRC patients.^43^ Elevated concentrations of another extracellular matrix-associated protein, MMP9, were also reported to be present in faeces of patients with this disease.^44^ However, in our present study, these markers were clearly less efficient for CRC detection than haemoglobin, especially in the ‘triage’ setting (see Fig. 4). Likewise, M2-PK, which was previously regarded as a promising faecal biomarker for CRC,^45,46^ performed modestly in this setting.

In the report on our previous study^30^, we highlighted PADI4 as an intriguing CRC biomarker possibly reflecting an increased probability of extracellular DNA trap formation^47,48^ in the mucus overlaying colorectal malignancies.^49^ The present study has, however, shown that  PADI4 allowed reliably distinguishing CRC patients from healthy volunteers, but failed to do so in the ‘triage’ setting. This suggests that the presence of elevated PADI4 levels in CM can be provoked by a wide range of gastrointestinal disorders, possibly including IBS, diverticulosis and small polyps.

The comparison of CM-associated biomarkers performed in this study allows concluding that haemoglobin measurement in CM provides high sensitivity and specificity values for CRC detection. Increased concentrations of CRP, TIMP1, M2-PK, MMP9 and PADI4 in CM samples could also serve as CRC biomarkers, but were less efficient in comparison with haemoglobin. In contrast, EGFR, calprotectin and EDN could not be recommended as reliable diagnostic markers. The presented results indicate that non-invasive CM self-sampling was very well accepted by the study participants and can be regarded as an attractive alternative to the collection of faeces. This point should be stressed, given that the necessity of collecting faecal samples may negatively affect compliance in CRC screening.^18,19^ In addition, study results suggest that this approach may present a very useful tool for triage of patients with abdominal symptoms to determine those who will benefit most from invasive colonoscopies.

The study had obvious limitations due to its relatively small size and the absence of direct comparison between our CM tests and FIT. Volunteers of the control group were considerably younger than the CRC patients; thus, our ‘screening setting’ results could potentially be biased. Moreover, we could not assess CM biomarker performance for colorectal polyp detection since sampling was performed post-colonoscopy, when the detected high-risk polyps were removed and only patients with very small polyps were included in the CRC-free symptomatic group. Finally, all analytical procedures were laboratory-based, but it is apparent that CM sample testing for haemoglobin can be easily presented as an inexpensive rapid point of care test, which remains to be developed and clinically evaluated.

All these points need to be addressed in our future work that could include larger prospective clinical studies addressing CRC screening and triage separately.

## Data Availability

All data generated by the study are included in the article.
